# The serological prevalence of SARS‐CoV‐2 infection in patients with chronic myeloid leukemia is similar to that in the general population

**DOI:** 10.1002/cam4.4179

**Published:** 2021-08-31

**Authors:** Massimiliano Bonifacio, Mario Tiribelli, Maria Cristina Miggiano, Elisabetta Abruzzese, Gianni Binotto, Luigi Scaffidi, Maddalena Cordioli, Daniela Damiani, Eros Di Bona, Malgorzata Monika Trawinska, Ilaria Tanasi, Maria Vittoria Dubbini, Vanessa Velotta, Giulia Ceccarelli, Elisabetta Pierdomenico, Mariella Lo Schirico, Gianpietro Semenzato, Marco Ruggeri, Renato Fanin, Evelina Tacconelli, Giovanni Pizzolo, Mauro Krampera

**Affiliations:** ^1^ Department of Medicine Section of Hematology University of Verona Verona Italy; ^2^ Division of Hematology and BMT Department of Medical Area University of Udine Udine Italy; ^3^ Hematology Department San Bortolo Hospital Azienda ULSS8 “Berica” of Vicenza Vicenza Italy; ^4^ Hematology, S. Eugenio Hospital ASL Roma2 Tor Vergata University Rome Italy; ^5^ Padua School of Medicine Department of Medicine, Hematology and Clinical Immunology Padua Italy; ^6^ Department of Diagnostics and Public Health Section of Infectious Diseases University of Verona Verona Italy

**Keywords:** chronic myeloid leukemia, COVID‐19, prevalence, serological tests, TKIs

## Abstract

**Background:**

Patients with hematological malignancies are at an increased risk of SARS‐CoV‐2 disease (COVID‐19) and adverse outcome. However, a low mortality rate has been reported in patients with chronic myeloid leukemia (CML). Preclinical evidence suggests that tyrosine kinase inhibitors (TKIs) may have a protective role against severe COVID‐19.

**Methods:**

We conducted a cross‐sectional study of 564 consecutive patients with CML who were tested for anti‐SARS‐CoV‐2 IgG/IgM antibodies at their first outpatient visit between May and early November 2020 in five hematologic centers representative of three Italian regions.

**Results:**

The estimated serological prevalence of SARS‐CoV‐2 infection in patients with CML after the first pandemic wave was similar to that in the general population (about 2%), both at national and regional levels. CML patients with positive anti‐SARS‐CoV‐2 serology were more frequently male (*p* = 0.027) and active workers (*p* = 0.012), while there was no significant association with TKI treatment type. Only 3 out of 11 IgG‐positive patients had previously received a molecular diagnosis of COVID‐19, while the remainders were asymptomatic or with mild symptoms.

**Conclusions:**

Our data confirm that the course of SARS‐CoV‐2 infection in patients with CML is generally mild and reassure about the safety of continuing TKIs during the COVID‐19 pandemic. Furthermore, we suggest that patients with CML succeed to mount an antibody response after exposure to SARS‐CoV‐2, similar to the general population.

## Graphical abstract

The estimated serological prevalence of SARS‐CoV‐2 infection in patients with CML after the first pandemic wave was similar to that of the general population (about 2% in Italy). Our data reassure about the safety of continuing TKI treatment during the ongoing pandemic and suggest that patients with CML succeed to mount an antibody response against SARS‐CoV‐2 whether on TKI treatment or not.

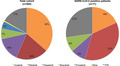

## 1 INTRODUCTION

The clinical course of the 2019 coronavirus (SARS‐CoV‐2) disease (COVID‐19) is extremely heterogeneous, with infected individuals being either asymptomatic or developing severe acute respiratory manifestations.^1^ Patients with hematological malignancies are at an increased risk of severe COVID‐19 and unfavorable outcome.^2^ However, comparing the relative frequencies of hematological disorders observed in COVID‐19 patients seen in March 2020 and in patients managed during 2019 at the same hematological centers revealed that patients with chronic myeloproliferative neoplasms, including chronic myeloid leukemia (CML) were underrepresented among hospitalized patients.^3^

Imatinib, the first generation tyrosine kinase inhibitor (TKI) has dramatically changed the history of CML treatment, increasing the rates of molecular responses, reducing the likelihood of natural progression of CML from chronic to advanced phases, and improving the survival.^4^, ^5^ Other BCR‐ABL1 inhibitors, namely nilotinib, dasatinib, bosutinib, and ponatinib, have been subsequently developed to overcome the resistance or intolerance to imatinib.^6^, ^7^, ^8^, ^9^ Still limited evidence suggests that some of these drugs may have a direct anti‐viral action of an indirect effect on the host response to SARS‐CoV‐2 infection, and ongoing trials aim at verifying the effect of TKIs in preventing pulmonary vascular leak in patients with severe COVID‐19 pneumonia.^10^

To date, the prevalence of SARS‐CoV‐2 infection in patients with CML has been reported through the collection of infected cases, as determined by molecular testing on symptomatic individuals and/or contacts through qRT‐PCR on pharyngeal swabs, and estimating the frequency over the total number of patients followed at each center, without direct evaluation of asymptomatic patients. The reported prevalence in these studies, conducted during the first months of pandemic, ranged from 0.17% to 0.9%.^11^, ^12^, ^13^, ^14^, ^15^

The seroprevalence of SARS‐CoV‐2 infection in a cohort of patients with CML, including symptomatic and asymptomatic individuals, has not been reported yet.

## 2 METHODS

### 2.1 Study procedures

In this experimental, cross‐sectional, non‐pharmacological study we enrolled all consecutive patients with CML attending five outpatient hematological centers in three Italian regions with different prevalence of infection in the general population (Veneto, Friuli‐Venezia Giulia, and Lazio). The inclusion criteria comprehended an established diagnosis of CML according to the World Health Organization criteria and age ≥18 years. No exclusion criteria have been envisaged, except for the unwillingness to sign a written informed consent. Patients enrolled in clinical trials were not excluded unless the participation to other experimental, non‐pharmacological studies was formally precluded by the study protocol. The study was approved by local IRBs.

After gathering the information about risk factors for COVID‐19 (travels, occupational exposure, living with infected individuals) and respiratory or general symptoms experienced from mid‐February 2020, patients were tested for anti‐SARS‐CoV‐2 IgM and/or IgG antibodies through a qualitative immunochromatographic assay (COVID‐19 IgG/IgM Rapid Test Cassette, Menarini Diagnostics, IT; sensitivity IgG 97.2%, IgM 87.9%, specificity IgG/IgM 100%). Patients with positive results underwent a nasopharyngeal swab for molecular detection of the virus.

### 2.2 Comparison group

The prevalence of SARS‐COV‐2 infection in the general population was retrieved from the Istat‐Italian Ministry of Health, SARS‐CoV‐2 seroprevalence study, which was conducted between May and July 2020 on 64,660 individuals, and estimated an anti‐SARS‐CoV‐2 IgG Italian prevalence of 2.5%, with marked regional differences ranging from 0.3% (Sicily and Sardinia) to 7.5% (Lombardy). As of 15 July 2020 the prevalence of SARS‐CoV‐2 infection in the three regions involved in the present study was 1.9 (95%CI 1.4–2,5) for Veneto, 1.0 (95%CI 0.6–1.5) for Friuli‐Venezia Giulia, and 1.0 (95%CI 0.6–1.3) for Lazio.^16^

### 2.3 Statistical analysis

Epidemiological and serological patterns were compared using the chi‐squared test for differences in terms of categorical variables (or Fisher's exact test when appropriated), *t*‐test or Mann–Whitney test (when appropriated) for difference in terms of continuous variables. For all hypotheses tested, two‐tailed *p* values < 0.05 were considered to be significant.

## 3 RESULTS

### 3.1 Study population

From 18 May 2020 to 3 November 2020 a total of 564 patients with CML were tested. None refused to participate. Patients enrolled in ongoing experimental clinical trials were all included in the present study since none of those study protocols formally precluded the participation to other experimental, non‐pharmacological studies. Males were 317 (56%), the median age at study entry was 63.6 (range 19.6–93.9) years, and the median time since CML diagnosis was 8.6 (range 0.1–33.7) years.

Sokal risk distribution was 41%, 37%, and 14% for L/I/H categories, respectively (8% not available). Nearly half of the patients were on the first‐line TKI treatment (n = 281, 49.8%), while the remainders were on second line (n = 131, 23.2%), third, or further line of treatment (n = 58, 10.3%), or in treatment‐free remission (TFR) (n = 94, 16.7%).

The type of TKI currently taken was imatinib (n = 205, 43.6%), nilotinib (n = 114, 24.1%), dasatinib (n = 85, 18.1%), bosutinib (n = 37, 7.9%), ponatinib (n = 26, 5.6%), or experimental (n = 3, 0.7%). The molecular level of response at study entry was MR^4^.^0^ or better (n = 356, 63.1%), MR^3.0^ (n = 130, 23.1%), MR^2.0^ (n = 35, 6.2%), or no response/recent diagnosis (n = 43, 7.6%).

### 3.2 Serological prevalence of SARS‐CoV‐2 infection

Eleven out of 564 patients had a positive IgG test and 3 of them were also IgM‐positive, for an estimated serological prevalence of SARS‐CoV‐2 infection in the CML population of 1.95% (95%CI 1.09–3.46), which was close to the national serological prevalence on the general population (Table 1). None of them had an active infection at the time of study, since all tested negative on the molecular nasopharyngeal swab performed immediately after the serological assay. Nine out of these 11 patients came from the Veneto region, one from Friuli‐Venezia Giulia, and one from Lazio; the estimated prevalence of infection in the three regions was 2.7%, 1%, and 0.8%, respectively, which was comparable to the general population also at the regional level.

**TABLE 1:** Characteristics of patients with positive serological results

Gender/age	Work	Contact with COVID−19 cases	SARS‐CoV−2 IgG/IgM	Previous diagnosis of COVID−19	Any symptoms	Time between symptoms/diagnosis and serological test (weeks)	Current TKI	Line of treatment
F / 71	Retired	No	Pos/pos	No	Yes	6	Bosutinib	2
M / 51	Employee	Yes	Pos/neg	Yes	Yes	12	Nilotinib	1
M / 74	Retired	No	Pos/neg	No	Yes	8	None (TFR)	n.a.
M / 60	Worker	No	Pos/neg	Yes	No	10	Dasatinib	2
M / 52	Worker	No	Pos/neg	No	Yes	6	Imatinib	1
M / 60	Manager	No	Pos/pos	No	Yes	4	Imatinib	1
M / 49	Worker	No	Pos/neg	No	No	n.a.	Nilotinib	1
M / 45	Employee	Yes	Pos/pos	Yes	Yes	3	None (TFR)	n.a.
M / 53	Worker	No	Pos/neg	No	Yes	11	Nilotinib	2
M / 31	Freelance	No	Pos/neg	No	Yes	9	Nilotinib	1
M / 44	Cook	No	Pos/neg	No	No	n.a.	None (TFR)	n.a.

### 3.3 Serological status and modality of CML treatment

**FIGURE 1:**
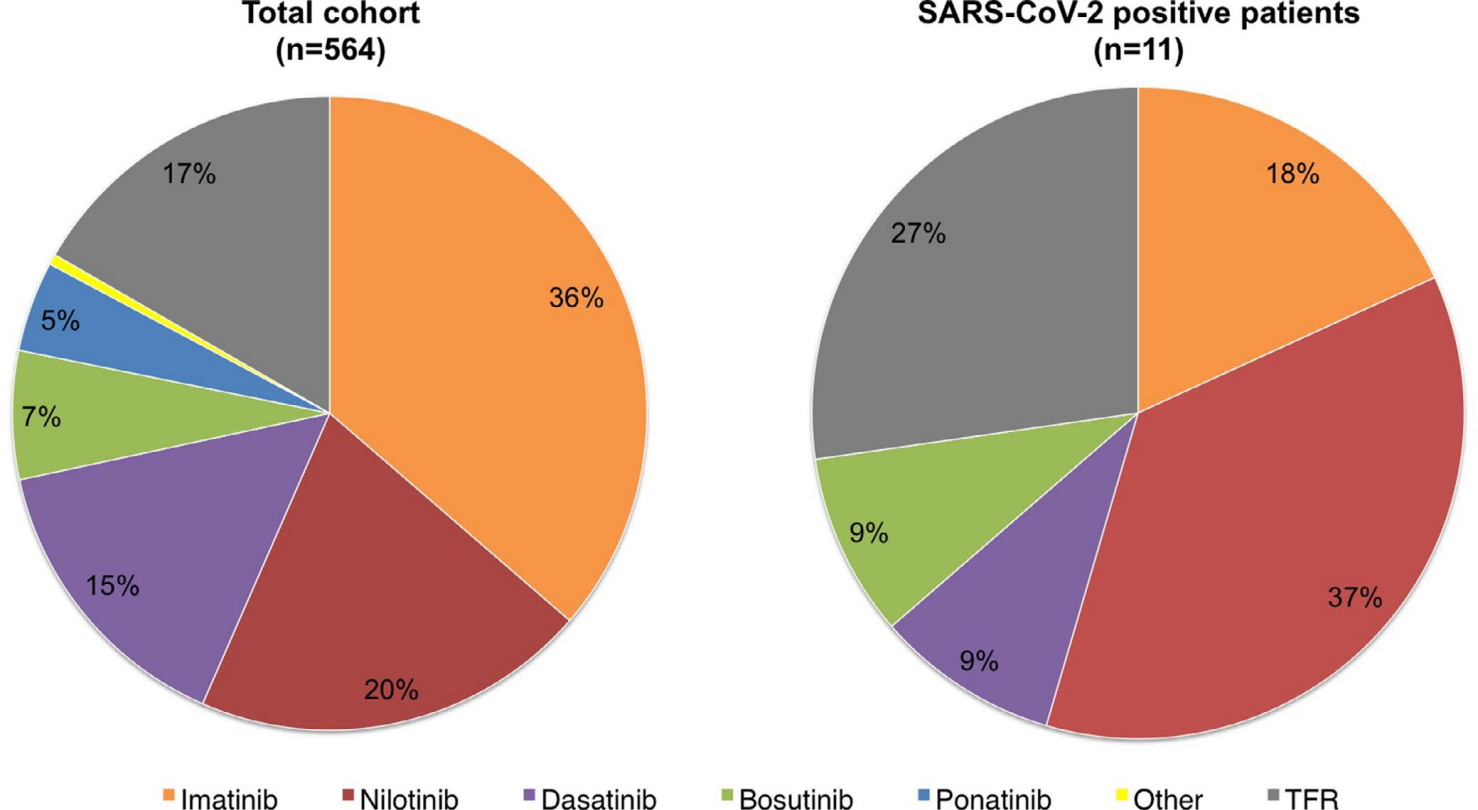
Treatment modalities (frequency of each TKI, or TFR status) in the total CML cohort (left panel) and in patients with positive anti‐SARS‐CoV‐2 serological test (right panel)

Eight of the 11 IgG‐positive patients were taking TKI treatment and 3 were not taking any TKI (i.e., patients in TFR) (Figure 1). There was no statistically significant association between positive serological test and the type of TKI treatment (*p* = 0.19), nor TFR status (*p* = 0.40).

### 3.4 Serological status and risk factors for SARS‐CoV‐2 infection

Active workers were 43% of the enrolled patients in the entire cohort, and the remainders were retired (42.5%), students (0.8%), or unemployed (13.7%). Nineteen, 4, and 1 patients reported close contact with COVID‐19 infected individuals at work, at home, or both, respectively.

Patients with positive anti‐SARS‐CoV‐2 serological test were more frequently male (*p* = 0.027) and active workers (*p* = 0.012). Only three patients were previously diagnosed as having COVID‐19, in two cases after close contact with infected subjects, while the other patients were asymptomatic or with mild symptoms and had not performed any diagnostic test for SARS‐CoV‐2 before. There was no statistically significant trend of association between positive serological test and contact with indices cases (*p* = 0.07).

### 3.5 Serological status and symptoms

Overall, the frequency of new onset or worsening symptoms reported during the last months was as follows: anosmia (2.3%), ageusia (1.8%), cough (5.3%), pharyngitis (4.3%), dyspnea (3.4%), fever (4.4%), headache (7.8%), asthenia (13.1%), arthralgia (13.1%), dizziness (6.2%), nausea/vomiting (2.7%), and diarrhea (4.8%).

The majority of these symptoms correlated with a positive anti‐SARS‐CoV‐2 serological test, and anosmia, ageusia, fever, asthenia, and arthralgia had the most significant association (Table 2).

**TABLE 2:** Correlation of new onset or worsening symptoms and serological status

Symptom	SARS‐CoV−2‐positive patients (N = 11) n. (%)	SARS‐CoV−2‐negative patients (N = 553) n. (%)	*p*
Anosmia	5 (45.5)	8 (1.5)	<0.001
Ageusia	5 (45.5)	5 (0.9)	<0.001
Cough	3 (27.3)	27 (4.9)	0.017
Pharyngitis	2 (18.2)	22 (4.0)	0.075
Dyspnea	0	19 (3.4)	ns
Fever	4 (36.4)	21 (3.8)	<0.001
Headache	2 (18.2)	42 (7.6)	0.21
Asthenia	5 (45.5)	69 (12.5)	0.008
Arthralgia	5 (45.5)	69 (12.5)	0.008
Dizziness	4 (36.4)	31 (5.6)	0.003
Nausea/vomiting	0	15 (2.7)	ns
Diarrhea	1 (9.1)	26 (4.7)	0.42

## 4 DISCUSSION

The prevalence of SARS‐CoV‐2 infection in patients with CML has been reported to date through the collection of infected cases, as determined by molecular testing on symptomatic individuals, and considering the total of patients with CML in charge as the denominator. In the first report from the Hubei Province, Li et al described 5 COVID‐19 cases out of 530 patients with CML (0.9%), with a higher prevalence in advanced CML or in subjects without complete hematologic response.^11^ In a Dutch study, 25% of patients with CML reported mild respiratory symptoms in a self‐assessment questionnaire, but confirmed molecular diagnosis was made in only one subject (0.7%).^13^ A study from Turkey showed that ICU admission rates, the need for mechanical ventilation, and mortality were lower in COVID‐19 patients with CML treated with TKI compared to age, gender, and comorbidity‐matched control patients without cancer.^14^ Finally, Breccia et al conducted a large survey on the centers belonging to the Italian Campus CML program, comprising 6,883 patients, with a low reported frequency of infection (0.17%) and a much higher impact of COVID‐19 pandemic in assuring regular monitoring, TFR strategies, and drug delivery.^12^

Differently from these studies, we collected comprehensive data on all patients enrolled in this unselected cohort, thus being able to compare the characteristics of infected and uninfected patients, including the type of TKI treatment. The study was promoted by the University of Verona and other two representative Venetian Centers were involved in the project since the Veneto region was one of the first areas in which autochthonous COVID‐19 cases appeared and rapidly spread at the beginning of pandemic. The other two hematologic centers, one in Friuli‐Venezia Giulia and one in Lazio, were chosen because they were regional reference centers for CML, and the prevalence of the infection in those regions during the first pandemic wave was different from that of the Veneto region.

The frequency of anti‐SARS‐CoV‐2 positive patients taking nilotinib was numerically higher than that of cases treated with other TKIs (Figure 1), but the difference did not reach statistical significance. Similarly, the percentage of IgG positive patients was higher in TFR (3.1%) than in TKI‐treated patients (1.7%), but the difference was not statistically significant.

In vitro studies and limited clinical evidence suggest that TKIs may have a direct antiviral action and/or indirect immunomodulatory effect.^10^ Imatinib and nilotinib were shown to prevent the fusion of SARS‐CoV and MERS‐CoV Spike (S)‐protein with the human epithelial cell membrane via Abl‐mediated cytoskeletal rearrangement.^17^ In addition, TKIs have been claimed to counteract viral growth through the inhibition of Abl2, a cell protein required for viral replication.^18^ Other putative anti‐coronavirus mechanisms of TKIs include improvement of pulmonary endothelial barrier dysfunction in acute lung injury,^19^ and an immunomodulatory role that may be protective against cytokine storm associated with advanced COVID‐19 infection.^20^ Consistent with these preclinical observations, the reported outcome of COVID‐19 infection in patients with CML was generally mild.^3^, ^14^, ^21^ Also in the present work, the majority of patients with CML had no or mild symptoms, and were not deemed worthy of screening during the first pandemic phase, when the access to molecular diagnostic tests was subjected to quota. On the other hand, to estimate the actual prevalence of anti‐SARS‐CoV‐2 antibodies in the entire CML population, we did not exclude from the study patients previously diagnosed as having COVID‐19 infection, and in all these three cases, our test confirmed the IgG‐positive status, as early as 3 weeks after the molecular diagnosis of infection.

The question remains whether TKI might prevent the infection by antagonizing the fusion of S‐protein with the cell membrane, or by inhibiting the intracellular replication of the virus, or they simply reduce the overproduction of inflammatory cytokines, which are ultimately responsible for organ failure. Due to the limited number of subjects exposed to SARS‐CoV‐2 in the general population after the first pandemic wave, in the present study we could not demonstrate a preventive role of TKI against infection. However, when limiting the analysis to the period (May–July) considered by the ISTAT study on Italian population, the prevalence on patients with CML appeared to be lower (1.06%). Instead, a contribution toward a milder course of the infection can be assumed due to the absence of seriously ill patients. Further studies are needed to ultimately define the TKI contribution against SARS‐CoV‐2, by comparing on a larger number of cases the seroprevalence of SARS‐CoV‐2 infection in patients with CML on TKI treatment and those in TFR.

We acknowledge the use of a rapid test as a potential limitation. However, rapid tests have been developed to allow extensive testing in conditions where venipuncture and lab infrastructures are not easily available, as happens during a pandemic. We also recognize that the choice of a single study center for the Friuli‐Venezia Giulia and Lazio regions may limit the generalization of our findings. However, these centers of reference for CML manage a high number of patients, and the comparison with the data of the Istat survey, which sampled around 0.1% of the entire Italian population, is legitimate.

Overall, our data show that prevalence of anti‐SARS‐CoV‐2 IgM and/or IgG positivity in patients with CML was comparable to the general population in the same period both at national and regional levels, and reassure about the safety of continuing TKI treatment in patients with CML during the ongoing SARS‐CoV‐2 pandemic. Furthermore, our data suggest that patients with CML, whether on TKI treatment or not, are able to mount an antibody response after exposure to SARS‐CoV‐2. This observation might imply that in patients with CML the efficiency of vaccination against SARS‐CoV‐2 could be similar to that of the general population, but this hypothesis needs to be confirmed by future research.

## 5 ETHICS APPROVAL STATEMENT

The study was reviewed and approved by the Ethics Committee of the coordinating Center (Comitato Etico per la Sperimentazione Clinica delle Province di Verona e Rovigo, ref. 2693CESC on 4 May 2020) and approved by the local IRBs of all the participating Centers.

## CONFLICT OF INTEREST

None of the authors have any conflicts of interest to report.

## AUTHOR CONTRIBUTIONS

MB and MT designed the study, analyzed and interpreted the data, and wrote the paper. GP designed and supervised the project. MK served as the principal investigator, MB and ET served as co‐principal investigators. MCM, EA, GB, LS, MC, DD, EDB, MMT, IT, MVD, VV, GC, EP, and MLS recruited participants, performed the study procedures, and collected and interpreted the data. GS, MR, and RF supervised the project. All authors contributed to the review of the paper. All authors agreed to be accountable for all aspects of the work in ensuring that questions related to the accuracy or integrity of any part of the work are appropriately investigated and resolved.

## Data Availability

The data that support the findings of this study are available from the corresponding author upon reasonable request.
